# Bond Strength of Universal Adhesive/Resin Cement Combinations Relying on Touch-Cure Mechanisms

**DOI:** 10.3390/polym17091224

**Published:** 2025-04-29

**Authors:** Annamaria Forte, Eugenia Baena, Claudia Mazzitelli, Edoardo Mancuso, Diego D’Urso, Gerardo Pellegrino, Laura Ceballos, Lorenzo Breschi, Annalisa Mazzoni, Tatjana Maravic

**Affiliations:** 1 Department of Biomedical and Neuromotor Sciences, University of Bologna, Via San Vitale 59, 40125 Bologna, Italy; annamaria.forte2@unibo.it (A.F.); diego.durso2@unibo.it (D.D.); gerardo.pellegrino2@unibo.it (G.P.); lorenzo.breschi@unibo.it (L.B.); annalisa.mazzoni@unibo.it (A.M.); tatjana.maravic@unibo.it (T.M.); 2 IDIBO Research Group, Nursing and Stomatology Department, Rey Juan Carlos University, Avda. Atenas s/n, 28922 Madrid, Spain; eugenia.baena@urjc.es (E.B.); laura.ceballos@urjc.es (L.C.)

**Keywords:** resin cement, universal adhesive, dual-curing, self-curing, bond strength, touch-cure polymerization, silane

## Abstract

New dual-curing resin cements are constantly launched into the market to improve the bond strength between dentine and indirect restorations when light irradiation is limited by the restoration material. The present study evaluated the microshear bond strength (μSBS) of two dual-cured resin cements, Estecem II Plus (EP) and Variolink Esthetic DC (VAR), when resin composite or dentine substrates were conditioned with their corresponding universal adhesives, Tokuyama Universal Bond II (TUB) and Adhese Universal DC (ADH). The experimental groups (*n* = 20) were (1) TUB/EP light-cured, (2) TUB/EP self-cured, (3) ADH/VAR light-cured, and (4) ADH/VAR self-cured. A μSBS test was performed after 24 h (T0) or after thermocycling (TC), and failure modes were assessed. Data analysis was performed using three-way ANOVA and Tukey tests (*p* < 0.05). In composite, TUB/EP self-cured demonstrated the highest μSBS at T0 and TC. After TC, TUB/EP self-cured and ADH/VAR light-cured remained stable (*p* > 0.05). In dentine, TUB/EP light-cured was statistically superior to TUB/EP self-cured and ADH/VAR self-cured at T0. Thermocycling decreased the μSBS of light-curing groups. TUB/EP achieved optimal μSBS when the manufacturer’s instructions were followed and the adhesive was self-cured, irrespective of the bonding substrate. However, ADH/VAR was more dependent on the type of bonding substrate than on the curing mode of the resin cement.

## 1. Introduction

Indirect partial restorations (IPR) are a common treatment in daily dental practice in cases where posterior teeth have suffered an extensive loss of structure [1,2], helping clinicians to achieve an optimal anatomy and contact point [3].

Their adhesive cementation is a crucial step to guarantee retention, adequate marginal adaptation, and longevity of the restorations [4,5]. To date, dual-cure resin composite cements have been considered the first choice to lute IPR. Indeed, photo-initiators and redox systems of dual-cure resin cements enable optimal polymerization regardless of the partial or total light transmittance attenuation related to the presence of the restorative material [6].

Resin cement systems that entail conditioning of the bonding substrates by means of universal adhesives (UAs) or primers prior to dual-cure resin cement application have been suggested to offer better retention and longevity of IPRs compared to self-adhesive cements [7,8]. UAs are the latest bonding system generation introduced to the market. These systems represent a mixture of ingredients added to guarantee reliable bonding performances along with ease of use, therefore gaining increased popularity among clinicians and educators thanks to the possibility of being applied in etch and rinse (EAR), self-etch (SE), or selective enamel etching (SEE) methods on tooth substrates and restorative materials, depending on the clinical context [9]. In general, UAs differ in terms of composition, even though 10-methacryloyloxydecyl dihydrogen phosphate (10-MDP) represents the most used acidic functional monomer across various adhesive formulations. This is not surprising since this specific functional monomer has demonstrated great tooth demineralization ability and particular interaction with calcium ions present in dentine. The resulting MDP-Ca salts ensure stronger and more stable bonds that are highly resistant to degradation [10,11]. On the other hand, some UAs may contain silanes to enhance the bonding interaction with different restorative materials (i.e., resin composites, ceramics etc.) [12,13].

In general, light irradiation has been considered fundamental to increase the polymerization reaction of dual-cure resin cements, as self-cure alone was not able to achieve comparable results. In order to improve the chemical curing reaction of these resin cements, a new polymerization concept, known as “touch cure” or “contact cure”, has been introduced to some UA systems. This technology has been claimed to enhance the chemical polymerization reaction once the accelerators of UAs come in contact with the resin cements, resulting in a more complete and stronger curing process [14,15]. Ideally, the touch-cure reaction would close the gaps existing in self-cure polymerization. It is noteworthy that strict adherence to manufacturers’ recommendations regarding material compatibility is essential to ensure the proper function of the proposed technologies and that deviation from these guidelines may disrupt the intended chemical reactions, potentially compromising their efficiency and the overall performance of the system [16]. However, little knowledge still exists on the mechanism beyond this curing modality, particularly with the most recently introduced adhesive systems.

Recently, a two-bottle (Bond A and Bond B; Tokuyama Dental Corp., Tokyo, Japan), silane-containing UA based on a touch-cure technology has been launched on the market. The thiol-functionalized γ-Mercaptopropyltriethoxysilane (MPTES) silane coupling agent (i.e., ϓ-PTS- gamma-methacryloxy propyl trimethoxy) contained in this UA has been described to be more stable than the traditional silane molecules present in most counterpart adhesives [17,18].

These characteristics are particularly warranted in those challenging cases where different substrates of adhesion can be present. Indeed, clinically, dentists usually have to bond IPR on a mixed substrate composed of the remaining dentine and the resin composite build-up restoration performed to relocate a subgingival cervical margin to a supragingival position or to improve the geometry of the preparation with a direct restoration [19].

Therefore, the aim of the present study was to evaluate the microshear bond strength (μSBS) of two different dual-cure adhesive resin cements in conjunction with their respective UAs on two substrates (resin composite and dentine) after artificial aging.

In order to test the effect of the variables (materials, curing mode, and aging) on the two different bonding substrates, the following hypotheses were tested: (1) no differences in bond strength exist among the two universal adhesive/resin cement combinations irrespective of the bonding substrate; (2) the curing mode does not influence the bonding performances of the tested materials, and (3) artificial aging does not influence bond strength.

## 2. Materials and Methods

**Figure 1:**
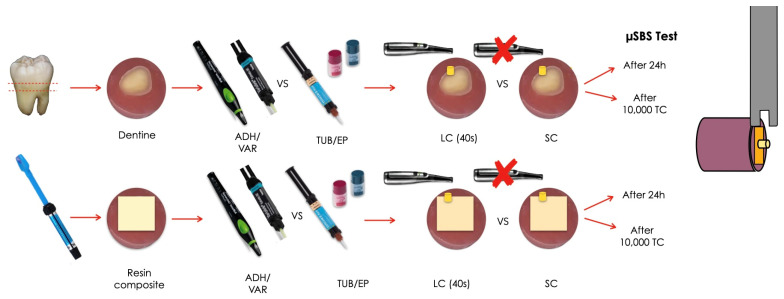
Schematic representation of the experimental set-up followed in the study. Two adhesive luting materials (Tokuyama Universal Bond II + Estecem II Plus—TUB/EP—and Adhese Universal + Variolink Esthetic—ADH/VAR) were used on dentine or resin composite substrates following different polymerization strategies (light-cure for 40 s—LC—and self-cure—SC). The retentive strength (μSBS) was evaluated after 24 h or 10,000 thermalcycles (corresponding to 1 year of clinical service).

Two different surfaces were used as bonding substrates: resin composite and dentine. A schematic representation of the study design is presented in Figure 1.

Eight resin composite blocks (15 × 15 × 4 mm^3^) were created with two 2 mm incremental layers of a microhybrid resin composite (Estelite Posterior, shade PA3, Tokuyama Dental Corp., Tokyo, Japan). Each layer was light-cured with a light-emitting diode (LED) curing lamp (Elipar Deep Cure-L, 3 M Oral Care, St Paul, MN, USA; output: 1.470 mW/cm^2^; wavelength: 430–480 nm). After complete polymerization, the specimens were stored in a laboratory oven at 37 °C for 24 h.

Regarding the second bonding substrate, 8 sound human molars were obtained from anonymous individuals following their informed consent under a protocol approved by the Ethical Committee of the University of Bologna, Bologna, Italy (protocol N°: 71/2019/OSS/AUSLBO). The crown of each tooth was cut 1 mm below the cemento-enamel junction with a diamond blade (Micromet, Remet, Casalecchio di Reno, Italy) under copious water cooling to remove the roots. Then, a second cut was made sagittally to expose coronal dentin. Each specimen was observed under an optical microscope at 50× magnification to check the absence of cracks and/or fractures.

Thereafter, both the resin composite and dentine blocks were embedded in an auto-polymerizing acrylic resin (Probase Cold, Ivoclar, Schaan, Liechtenstein), making sure not to touch or contaminate the surface available for bonding procedures. After complete setting of the resin, a smear layer was created on the dentine or composite with #600-grit SiC paper (Remet Polisher, Remet, Casalecchio di Reno, Italy) under water cooling to simulate clinical preparation with burs.

Further, a Teflon ring mold (1.84 × 3 mm^2^) was used to produce resin composite (Estelite Posterior, Tokuyama Dental Corp.) cylinders (*n* = 20 per group and per aging time). The cylinders were polymerized for 40 s from each side using the LED curing lamp (Elipar Deep Cure-L, 3 M Oral Care). After 24 h, the cylinders were polished for 30 s with a wet #600-grit SiC paper (Remet Polisher, Remet) to obtain a flat cementation surface. The cylinders were then distributed randomly to different groups and cemented to the two substrates as described in the following paragraph.

Dual-cured adhesive resin cements were employed for luting procedures with their respective adhesive systems: Estecem II Plus (EP) with the self-cured Tokuyama Universal Bond II adhesive (TUB; Tokuyama Dental Corp.) and Variolink Esthetic DC (VAR) with dual-cured Adhese Universal DC adhesive (ADH; Ivoclar, Schaan, Liechtenstein). Each resin cement was used either in light-cure (LC) or self-cure (SC) modes. The materials were handled strictly following manufacturers’ instructions and all the details of their compositions and application modes are presented in Table 1. Excesses of the cements were meticulously removed with a dental explorer and then the material was cured according to each experimental group. At the completion of luting procedures, the specimens were stored at 100% humidity and 37 °C to allow the complete setting of the resin cements.

Therefore, the following 4 groups were formed per bonding substrate, according to the adhesive procedures and curing mode of the resin cement (*n* = 20/per group/bonding substrate): (1) TUB/EP LC; (2) TUB/EP SC; (3) ADH/VAR LC; (4) ADH/VAR SC.

Once the specimens were prepared, one half were tested after 24 h of storage (T0), while the second half were submitted to accelerated aging through 10,000 thermocycles between 5–55 °C (THE100; SD Mechatronik, Westeham, Germany; dwell time 3 s and 5 s per each bath; TC). After their respective storage times, a microshear bond strength test (μSBS) using UltraTester (Ultradent Products, South Jordan, UT, USA) was executed. The shear load was applied at a cross-head speed of 0.5 mm/min perpendicularly to the cylinder/bonding interface until debonding. The bond resistance was registered in newtons (N) and divided by the bonded area to calculate the strength in megapascals (MPa).

After debonding, a failure analysis was conducted with a digital microscope at 100× magnification (VHX-7000, Keyence Corp, Osaka, Japan) on both the resin cement cylinder and the bonding substrate. Therefore, fracture modes were considered as follows: adhesive (A; at the cement/composite or dentin interface), cohesive (C; within the resin cement), and mixed (M; A and C occurring simultaneously).

Finally, statistical analysis was performed. Data were normally and equally distributed (Shapiro–Wilk and Brown–Forsythe tests *p* > 0.05, respectively). A three-way analysis of variance (3-way ANOVA) was conducted for each type of substrate to evaluate the influence of the independent variables (resin cement, curing mode, and aging) and their interactions on the dependent variable (μSBS). Then, pairwise multiple comparisons were performed using a Tukey test. Statistical significance was set at *α* = 0.05 for all analyses (SigmaPlot 14.0; Systat Software Inc., Berkshire, UK).

## 3. Results

### 3.1. μShear Bond Strength (μSBS) Testing

The μSBS (mean and standard deviation) for each experimental group on the two bonding substrates at T0 and TC are shown in Table 2.

#### 3.1.1. μShear Bond Strength to Resin Composite Substrate

Regarding the groups tested on the resin composite substrate, the three-way ANOVA revealed the influence of the independent variables—aging (*p* < 0.001), resin cement (*p* = 0.002), curing mode (*p* = 0.038), resin cement–curing interactions (*p* < 0.001), and aging–resin cement–curing mode interactions (*p* < 0.001)—on the dependent variable μSBS.

At T0, no differences were evidenced between TUB/EP LC, TUB/EP SC, and ADH/VAR LC (*p* > 0.05). In contrast, ADH/VAR SC attained the lowest bond strength among the groups (*p* < 0.05).

TC statistically decreased the bond strength for TUB/EP LC (*p* < 0.001) and ADH/VAR SC (*p* = 0.009), while no influence was evidenced for TUB/EP SC (*p* > 0.05) and ADH/VAR LC (*p* > 0.05). Among the TC groups, TUB/EP SC yielded the highest bond strength values among the groups (*p* < 0.05), while TUB/EP LC and ADH/VAR SC yielded the lowest (*p* < 0.05).

#### 3.1.2. μShear Bond Strength to Dentine Substrate

On those groups performed on dentine, the three-way ANOVA detected the influence of variable aging (*p* < 0.001), aging–resin cement interaction (*p* = 0.003), and aging–curing mode interaction (*p* < 0.001) on the variable μSBS.

After 24 h (T0) of the bonding procedures, the LC groups attained the greatest bond strength, with TUB/EP LC recording the highest values among the groups (*p* < 0.05). On the contrary, when the resin cements were SC, lower bonding values were registered, with no differences between TUB/EP SC and ADH/VAR SC (*p* > 0.05).

Artificial aging (TC) statistically affected the bond strength of the LC resin cement groups (TUB/EP LC *p* < 0.001; ADH/VAR LC *p* = 0.014), and this was particularly evident for TUB/EP LC, which attained the lowest μSBS values among the thermocycled groups (*p* < 0.05). Instead, at TC, ADH/VAR SC achieved the highest bonding values (*p* < 0.05), even though no differences could be observed with ADH/VAR LC and TUB/EP SC.

### 3.2. Failure Mode Analysis and Microscopy

The failure mode distribution for each experimental group, expressed in percentages, is shown in Table 3.

When analyzing the fracture modes of specimens luted on the resin composite substrate, mixed failures at T0 were the most common. After TC, adhesive and mixed failures occurred, with adhesive debonding being prevalent in the LC groups, irrespective of the luting system examined. No cohesive failures were registered in any group, either at T0 or after artificial aging.

On the dentine substrate, adhesive and mixed failures were present at T0. After thermocycling, mixed failures were the most prevalent for all groups except for ADH/VAR SC, in which mixed and adhesive failures were equally distributed. No cohesive fractures occurred in any groups or timepoints.

**Figure 2:**
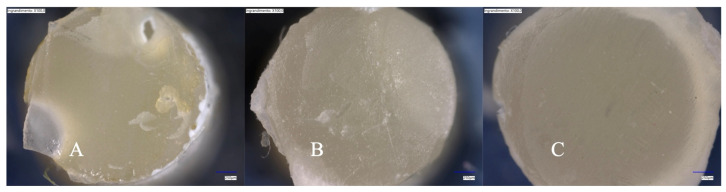
Representative digital microscope images (100× magnification) of the resin cement cylinders after debonding. (**A**): Adhesive fracture at the resin cement/bonding substrate interface (the luting material mass was completely attached to the composite cylinder side; (**B**): mixed fracture; (**C**): adhesive fracture at the resin cement/composite cylinder interface (the cylinder surface was completely free from resin cement residues).

Representative digital microscope images of the resin cement cylinders after debonding are presented in Figure 2.

## 4. Discussion

In the present study, the bonding performances of two different adhesive/resin cement combinations relying on touch-cure mechanisms after 10,000 thermal cycles, corresponding to 1 year of clinical service, were evaluated when luted on resin composite or dentine substrates. Based on the obtained results, all the tested hypotheses had to be rejected since the type of luting system, the curing mode, and the artificial aging influenced the retentive strength, irrespective of the bonding substrate of adhesion.

**Figure 3:**
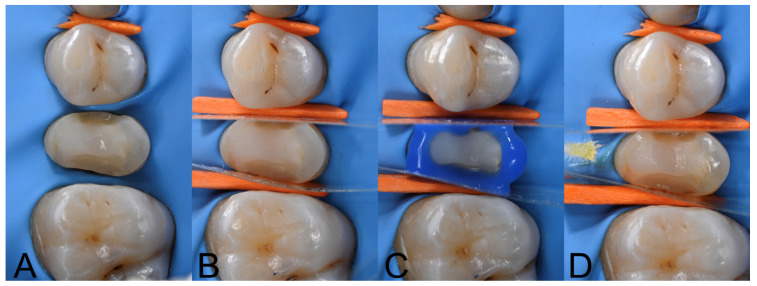
Clinical case illustrating the restorative and adhesive procedures performed prior to onlay cementation. In such cases, it is common to encounter multiple adhesive substrates, such as enamel and/or dentin and resin composite used for build-up reconstruction. Therefore, the adhesive system must ensure consistent clinical performance across both substrates. (**A**): Resin composite build-up on tooth #1.5; (**B**): protection of adjacent teeth before adhesive procedures; (**C**): after sandblasting of the resin-composite build-up, the enamel was etched with 37% phosphoric acid; (**D**): adhesive system application following manufacturer’s instructions.

The resin composite and dentine were considered as the bonding substrates to test the adhesive strength of the investigated luting systems. This choice relied on clinical background, as during restorative and prosthetic rehabilitations (i.e., IPR or core-build-up reconstructions) it is not unusual to encounter a combination of surfaces due to the resin composite build-up and the residual tooth structure (Figure 3) [19].

Therefore, limiting adhesion to a single substrate may be clinically restrictive in specific cases.

When luting on the resin composite substrate, at T0, the luting system TUB/EP, in both the LC and SC modes, yielded higher bond strength, as did ADH/VAR LC. In the case of the ADH/VAR combination, the curing mode was a determinant factor causing a significant drop in bond strength values, with the SC modality registering the lowest data among the T0 groups.

Although both universal adhesive/resin cement combinations incorporate a “touch-cure” or “contact-cure” technology, the mechanisms are different and may have possibly influenced the results. Indeed, Tokuyama Universal Bond II (TUB) is a self-curing two-bottle UA with an innovative curing mode consisting of the incorporation of a 3D-SR monomer and borate salts that generate free radicals to improve the adhesive SC polymerization even in the absence of light [20]. Previously, it has been demonstrated that the incorporation of borate salts for self-curing adhesives improved their degree of carbon-to-carbon (C=C) conversion [21] and, ultimately, the overall bonding values. In addition, the polymerization of the resin cement is enhanced when it contacts with the UA, finding an overall improvement of the degree of conversion compared to the resin cement itself without the activator application [22,23]. On the contrary, Adhese Universal DC (ADH) is a dual-curing adhesive which incorporates a co-initiator on the tip of the adhesive applicator. Although the type of co-initiator is not clearly stated by the manufacturer, a recent report [22] observed under SEM-EDS the presence of vanadium oxide particles on the bristle of an unused applicator, which indicates that the co-initiator mechanism is based on the generation of transitional metal salt accelerators. If, from one side, this mechanism improves the self-curing activity [22], and consequently the degree of conversion of ADH/VAR, nevertheless it was less effective than TUB (Table 2).

The literature argues that chemical polymerization alone worsens the mechanical properties of a polymeric material compared to LC mode [24]. The SC mechanism has been described to proceed slowly, requiring a certain amount of time to be completed [25,26]. During this timeframe, any adverse effect may compromise the overall reaction. Moreover, if the initial polymerization rate is too low, premature occlusal function may compromise the longevity of the restoration due to a decrease in bond strength and increases in monomer elution and solubility of the cement [22,27,28,29]. On one hand, the ADH/VAR combination obtained higher μSBS values, both on the composite and dentine, when in LC mode compared to SC mode. This is in concordance with a previous report which found that light curing increased the degree of conversion (DC) of several dual-cured resin cements combined with their universal adhesive when LC was performed [23]. Specifically, ADH/VAR LC resulted in DC 67.3% ± 2.2 compared to the 60.4% ± 1.7 of ADH/ VAR SC [22]. On the other hand, TUB/EP has been demonstrated to slowly increase its DC up to 24 h attaining high values regardless of the curing mode [23]. This translates into the assumption that, if the manufacturer’s instructions are strictly followed, SC accompanied by a touch-cure technology may work as efficiently as the LC modality in the case of a TUB/EP luting system.

In general terms, we found a tendency of lower μSBS and higher percentage of adhesive failures on dentine than on the composite substrate for all experimental groups. This highlights the difficulty of bonding to dentine, a matter of concern since the beginning of the era of adhesive dentistry [30]. Dentine is a heterogenous substrate which contains approximately 45 vol% mineral content (hydroxyapatite) and 33 vol% organic content, with the rest being water [31]. While hydrophilicity is desirable in the first instance for monomer diffusion within dentine, after solvent evaporation hydrophobicity is required to minimize hydrolytic degradation over time [32]. Initially, solvents play an important role in helping the adhesive to penetrate, removing the excess moisture of dentine efficiently [33]. According to the manufacturer’s information, ADH contains ethanol while TUB contains both ethanol and acetone as solvents (Table 1). The additional presence of acetone in TUB may help to eliminate the residual water quickly, because this solvent has been observed to evaporate faster than ethanol [33]. Presumably, this is a desirable effect on TBU to ensure that the slow initial SC process previously described [23] may be tolerant to the presence of wet and permeable substrates such as deep dentine.

The complicated bonding mechanism can have, however, multifactorial interferences, of which the adhesive formulation is among the most prominent ones. Both adhesives tested incorporate functional monomers within their composition. ADH contains 10-MDP, which is highly reactive and chemically interacts with dentine, creating calcium salts, resulting in a more stable bond over time [34]. On the other hand, TUB contains a novel 3D-SR functional monomer, which has several functional groups that can simultaneously interact with hydroxyapatite on one side and with other monomers on the other side (commercial literature). In SC mode, these monomers can have a longer interaction with dentine and possibly obtain a stronger chemical bond. In fact, in our research, self-curing groups kept their SBS stable on dentine even after aging (Table 2).

Another distinctive component among the tested adhesives is the type of silane present in each product. Silane interacts with silica-based materials through the hydroxyl groups, and the organofunctional monomeric ends of silane enable the reaction with methacrylate groups in the resin cement, providing additional adhesion [35]. Thus, it has been observed that the type of coupling agent may influence the stability of the adhesive/resin cement [36]. In this direction, TUB incorporates MPTES, considered a silane coupling agent with high stability over time. Conversely, ADH does not contain a silane within its formulation. However, these differences did not seem to influence the results of the present study, and the results were more dependent on the curing mechanism previously described. Although the silane can bind to the inorganic fillers of a resin composite, its influence on bond strength and longevity might be more influential for adhesion to ceramics. For instance, the preservation of bond strength values has been observed when the TUB/EP system was used to bond to lithium disilicate restorations after artificial aging [17].

## 5. Conclusions

Within the limitations of this in vitro study, the bonding properties of different dual-cure adhesive resin cements seem to be product-dependent, and their efficiency over time depends predominantly on the curing mode. Moreover, resin cements can perform differently depending on the substrate (composite vs. dentine). These differences should be clinically considered when luting to multi-material substrates.

Regarding the TUB/EP system:

(1) It demonstrated similar bonding performance across different curing modes when bonded to composite resin.
(2) In dentine, light curing increased the initial bond strength.
(3) Self-curing provided stable bond strength after aging.

Regarding the ADH/VAR systems:

(4) It benefited from light curing when bonding to the composite resin.
(5) In dentine, the bond strength remained stable with chemical polymerization.

## Figures and Tables

**Table 1:** List of the materials used. The table reports all details including the compositions and application modes as used in the study according to the manufacturer’s information.

Product Name (CODE). Shade. Manufacturer	Composition	Batch Number	Application Mode
ESTECEM II Plus (EP) Universal shade Tokuyama Dental Corp.	Paste A: Bis-GMA ^1^. TEDGMA ^2^. Bis-MPEPP ^3^, peroxide, camphorquinone, silica-zirconia filler (filler load 74% wt) Paste B: Phosphoric acid monomer, Bis-GMA ^1^, TEGDMA ^2^, HEMA ^4^, MTU-6 ^5^, silane coupling agent, peroxide. borate catalysts. acetone. ethanol. purified water.	A055BM	Apply with a mixing tip to the surface restoration. Seat the restoration within 1 min. Remove excess with a brush tip. Light cure for 20 s or more if translucent restorations. Light cure the margins for 20 s or more and allow to set for 8 min.
Variolink Esthetic DC (VAR) Neutral shade Ivoclar	UDMA ^6^, methycrilates, ytterbium trifluoride, spheroid mixed oxide, initiators, stabilizer, pigments and other ingredients	Z02YW3	Apply with a mixing tip to the surface restoration. Seat the restoration. Remove excess. Cover the margins with glycerine. Photopolimerize (10–20 s x mm ceramic and segment).
Tokuyama Universal Bond II (TUB) Tokuyama Dental Corp.	Phosphoric acid monomer (new 3D-SR monomer ^7^), Bis-GMA ^1^, TEGDMA ^2^, HEMA ^4^, MTU-6 ^5^, silane coupling agent. Peroxide, borate catalyst, acetone, ethanol and purified water.	Bond A: (003M1) Bond B: (502BM1)	Mix one drop of bond A and bond B into mixing well or disposable mixing well until it turns green. Apply within 1 min (mixing well) or 3 min (disposable mixing well). Air dry within 30 s of application.
Adhese Universal DC (ADH) single dose (Ivoclar)	Methacrylates, ethanol, water, dispersed silicon dioxide, initiators, stabilizers. Applicator coated with co-initiators.	Z027G5	Remove the green applicator brush from tis chamber. Mix the adhesive with the co-initiator for 5 s by twisting the applicator until it turns yellow. Scrub the surface for 20 s. Once activated, the adhesive can be used for approx. 120 s. Air dry until glossy, immobile film layer results. Polymerization: light cure for 10 s or self-cured if combines with VAR.
Estelite posterior PA3 shade (Tokuyama Dental Corp.)	Bis-GMA ^1^, TEGDMA ^2^, Bis-MPEPP ^3^, 84% wt silica-zirconia filler (mean particle size: 2 μm, particle size range: 0.1 to 10 μm). Radical-Amplified Photopolymerization initiator technology (RAP)	W3723	Place on increments 1.8–2 mm. Photopolymerize 5–10 s if using a LED unit

^1^ Bis-GMA: Bisphenol A di(2-hydroxy propoxy) dimethacrylate; ^2^ TEGMA: Triethylene glycol dimethacrylate. ^3^ Bis-MPEPP^:^ Bisphenol A polyethoxy methacrylate. ^4^ HEMA: 2-Hydroxyethyl methacrylate. ^5^ MTU-6: thiouracil monomer. ^6^ UDMA: urethane dimethacrylate. ^7^ 3D-SR monomer: three-dimensional self-reinforcing monomer.

**Table 2:** Mean and standard deviation of μSBS expressed in MPa for each experimental group.

Experimental Group	Composite		Dentine	
	T0 $\bar{x}$ (SD)	TC $\bar{x}$ (SD)	T0 $\bar{x}$ (SD)	TC $\bar{x}$ (SD)
TUB/EP LC	29.3 (5.8) AB,a	17.0 (5.7) C,b	31.2 (12.6) A,a	8.8 (4.4) B,b
TUB/EP SC	33.2 (7.5) A,a	32.7 (7.5) A,a	20.5 (6.7) B,a	13.3 (6.9) AB,a
ADH/VAR LC	27.4 (6.9) AB,a	25.9 (5.9) B,a	23.3 (8.6) AB,a	12.5 (4.6) AB,b
ADH/VAR SC	26.3 (7.7) B,a	17.4 (7.8) C,b	19.5 (7.4) B,a	19.1 (4.7) A,a

Different capital letters in columns indicate significant differences (*p* < 0.05) among groups at each testing time. Different small letters in rows indicate statistically significant differences (*p* < 0.05) between T0 and TC in each experimental group for each substrate.

**Table 3:** Failure mode distribution expressed in % for each experimental group at each time (T0 and TC).

		Composite	Dentine
Experimental Group	Aging	A/C/M	A/C/M
TUB/EP LC	T0	0/0/100	50/0/50
	TC	15/0/85	69/0/31
TUB/EP SC	T0	0/0/100	67/0/33
	TC	7/0/93	27/0/73
ADH/VAR LC	T0	0/0/100	47/0/53
	TC	0/0/100	80/0/20
ADH/VAR SC	T0	0/0/100	73/0/27
	TC	47/0/53	53/0/47

A: adhesive failure; C: cohesive failure; M: mixed failure.

## Data Availability

Data are contained within the article. Further inquiries can be directed to the corresponding author.
